# Flexible hardware for teaching and research in renewable energy and off-grid microgrids

**DOI:** 10.1016/j.ohx.2025.e00636

**Published:** 2025-03-18

**Authors:** Cristian Escudero-Quintero, Juan Pablo Guzman-Rodriguez, Juan Pablo Villegas-Ceballos, Elkin Edilberto Henao-Bravo, Daniel Gonzalez-Montoya

**Affiliations:** Facultad de Ingeniería, Insitución Universitaria ITM, Medellín, 050041, Colombia

**Keywords:** Control of switching converters, Power converters, Power electronics, Inverters, Renewable energy

## Abstract

Microgrids are an active research field due to their integration of renewable and non-renewable energy sources alongside energy storage systems to supply both DC and AC loads. While existing studies extensively explore hierarchical control structures, focusing on algorithm design and optimization, practical implementation remains challenging due to system complexity. This paper presents the development of a low-power microgrid hardware platform based on power converters and controllers, enabling experimental validation of the second and third levels of hierarchical control. The platform enables real-time energy management using FPPT algorithms while providing an intuitive graphical interface for data visualization and system monitoring. Experimental results confirm the system’s capability of dynamically managing energy flows, ensuring stable operation under varying load conditions. The system demonstrates reliable operation handling loads of up to 100 W, with scalability to 200 W, providing an effective tool for testing and research in microgrid control strategies.

## Graphical abstract

**Figure:**
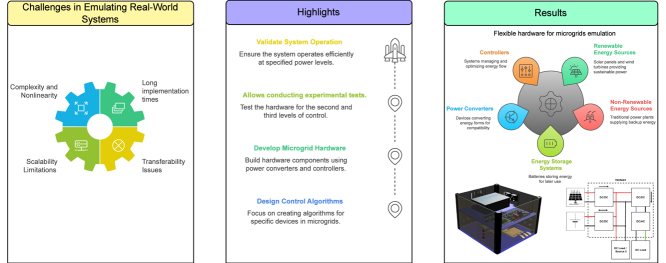

## Specifications table

**Table:** 

Specifications table	

Hardware name	Flexible hardware for teaching and research in renewable energy and off-grid microgrids.

Subject area	•Engineering and material science
	•Educational tools and open source alternatives to existing infrastructure
	•General
Hardware type	•Electronics engineering and computer science
Closest commercial	•Solar Micro Inverter Development Kit
analog	•Solar MPPT DC/DC development Kit
	•Solar Development Kit with PMIC
	•MPC5775B/E Low-Cost BMS and Inverter Development Board
	
Open source license	CERN Open Source License Permissive (CERN-OHL-P)
Cost of hardware	Approximate hardware cost: 416.13 USD
Source file repository	https://doi.org/10.17605/OSF.IO/PCMHW

	

## 1 Hardware in context

Microgrids constitute a dynamic and contemporary field of research, integrating various energy sources—both renewable and non-renewable to supply DC and AC loads. Some microgrids also incorporate energy storage devices to optimize the energy generated by renewable sources [1]. Additionally, they can operate either on-grid or off-grid, with off-grid systems being particularly relevant in areas where access to the main grid is limited or non-existent [2].

To ensure efficient and reliable operation, a three-level hierarchical control system is implemented in microgrids, with each level performing specific functions related to security, coordination, and efficiency [3]. The first level controls individual devices within the microgrid, such as inverters, regulators, and batteries, performing fine-tuning and local regulation to maintain stability and efficiency. The second level is responsible for local microgrid management, monitoring and adjusting power flow to meet demand by controlling the operation of generation, storage, and load devices. Finally, the third level oversees and coordinates the entire microgrid, ensuring proper grid operation while balancing resources and energy flows.

In the literature, extensive research covers various aspects of hierarchical control structures, focusing on designing and optimizing control algorithms for specific devices such as inverters, batteries, and storage systems. Moreover, adaptive control techniques have been developed to respond to variations in both demand and generation [4]. Likewise, the interaction between microgrids and the main grid is being studied to enhance operational efficiency.

Each of these studies requires validation of the proposed methodologies, techniques, or algorithms. However, due to the complexity of these systems, their practical application presents significant challenges. To address this, researchers validate their contributions through simulations. For example, [5] focuses on improving the efficiency of photovoltaic systems under partial shading conditions using a bio-inspired algorithm, while [6] explores energy management optimization in microgrids with storage, aiming to reduce costs and enhance system stability.

Moreover, validation through simulations on specialized platforms such as Hardware-in-the-Loop (HIL) has become a key tool to ensure the effectiveness of proposed algorithms and strategies before their implementation in real systems. In this regard, [7], [8] demonstrate the usefulness of HIL for evaluating control strategies in hybrid and islanded AC microgrids, respectively. Likewise, [9] employs real-time simulation with Typhoon HIL to test the control of a microgrid based on solar energy and battery storage. In contrast, [10] introduces the concept of a digital twin as an advanced tool for managing microgrids in autonomous vehicles.

Despite the accuracy of these methods to emulate the operation of systems in controlled environments, they often fail to cover all the complexities and nonlinearities of the real world. This limitation can lead to inaccuracies or the omission of relevant aspects, generating results that only partially reflect what happens in real situations [11]. In addition, the setup and development of these test environments often demand a significant amount of time and resources. Researchers must spend considerable effort to create accurate models and carry out complex simulations. Also, the results obtained in these controlled settings may not readily apply to larger situations or large-scale operating systems due to scalability or transferability limitations.

An adequate test scenario for evaluating algorithms and new power converters in a microgrid based on renewable energy sources is to perform tests in a real environment. However, given the risks and the high costs associated with this option, a more feasible alternative is to conduct the tests in a scaled system. In this context, it is essential to facilitate the interaction of the three levels of control, including the complexities inherent to the system and the variability inherent to renewable energy sources. In addition, testing should be run over realistic periods, ranging from hours to days, and specifically addressing the communication latencies between the different levels of the system.

In recent years, open-source hardware and software used in microgrids have gained significant relevance due to their flexibility, low cost, and adaptability in academic and research environments. [12] propose a consensus-based distributed hierarchical strategy for battery energy storage systems (BESS) in DC microgrids. This strategy employs an adaptive proportional consensus algorithm that efficiently allows state-of-charge (SOC) balancing and current sharing, improving microgrid stability and performance without needing a centralized controller. Similarly, [13] developed a low-cost, web-based SCADA system for a microgrid testbed. Using open platforms such as Arduino and Raspberry Pi, this system facilitates data acquisition and monitoring in hybrid microgrids that integrate solar, wind, and biomass sources and battery storage. This approach provides an economical and flexible solution for teaching and research, reducing the costs associated with commercial SCADA systems. In addition, [14] proposes an innovative multi-level architecture for automating and monitoring smart photovoltaic microgrids. The system uses the open Modbus TCP protocol to facilitate industrial and open-source equipment interoperability. The architecture has been experimentally validated in a photovoltaic microgrid, demonstrating its effectiveness in integrating diverse heterogeneous devices and systems.

Several commercial alternatives with flexible and modular structures adapt to different conversion schemes. The Solar Micro Inverter Development Kit [15] includes a flyback DC/DC converter with active clamping and an inverter that operates in both on-grid and off-grid modes, all digitally controlled by the TMS320F28035 Piccolo microcontroller; the platform is ideal for microinverters control research. The Solar MPPT DC/DC Development Kit [16] offers a two-phase boost converter with MPPT and an LLC resonant converter, complementing the Solar Inverter Kit [17], which offers a four-switch inverter controlled by the microcontroller TMS320F28035 Piccolo or F28M35H52C Concerto, and is suitable for central and string inverters. In addition, the Solar Explorer Development Kit serves as a low-voltage learning platform for solar application development.

On the other hand, the Solar Development Kit with PMIC from e-peas and supercapacitors from CAP-XX (DEV-EPEAS-CAPXX) [18] allows the integration of solar energy into electronic devices, using solar panels from PowerFilm and supercapacitors from CAP-XX. The MPC5775B/E Low-Cost BMS and Inverter Development Board [19] is designed for battery and inverter management, with 32-bit Power architecture MPC5775B/E microcontrollers and the MC3377x cell controller, being suitable for battery management, diesel engines, motorcycle engine control units and small motors. Finally, the PELab system includes an industrial-grade embedded controller with a dual-core ARM Cortex M7/M4 microcontroller and touch screen, offering flexible configurations for power electronics applications. Table 1 presents a summary of the hardware characteristics with capabilities and cost.

**Table 1:** Microgrid test hardware comparison.

Hardware	Power inputs	Possibility to connect more sources	Charger/discharger energy storage	Inverter	Cost (USD)
Solar Micro Inverter Development Kit	1	not	not	yes/ On-grid	$996

Solar MPPT DC/DC Development Kit	1	not	not	yes/ On-grid	$700

Solar DC/AC Inverter Kit,	1	not	not	yes/ On-grid	Obsolete

Solar Explorer Development Kit	1	not	yes	yes/ On-grid	Obsolete

Solar Development Kit con PMIC	1	not	yes	not	$80

MPC5775B/E Low-Cost BMS and Inverter Development Board	1	yes	yes	not	$279

**Proposed Hardware**	**2**	**yes**	**yes**	**yes/ Off-grid**	**$416.13**

## 2 Hardware description

The system design follows the usability and simplicity principles to facilitate its use in educational spaces, allowing students and teachers to interact with its different components without requiring advanced technical knowledge in hardware implementation. The user interface is intuitive and graphical through Simulink, providing real-time visualizations of variables of interest for the application. Additionally, the developers designed the interface and controls to guide users step by step in configuring and operating the system, enabling the creation of an easy-to-use interface in educational environments.

The hardware design simplifies physical connections between the different modules. Each element includes connection diagrams, allowing users to generate configurations that lead to new control and energy management strategies. In terms of software, the system integrates an interface that enables users to interact with the data and control the system through Simulink blocks, which is especially useful in academic contexts where the goal is to explain renewable energy principles and microgrid control.

**Fig. 1:**
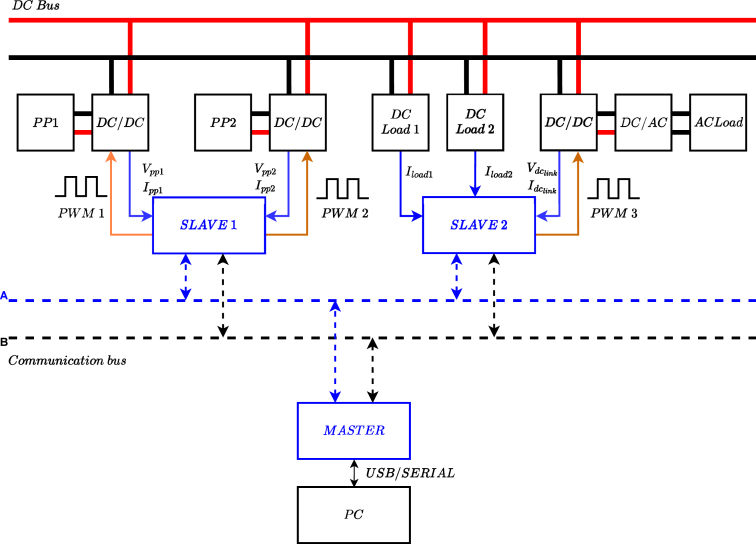
Proposed microgrid topology.

Looking to observe the behavior of a DC microgrid operating with different control techniques and management algorithms under controlled conditions, this work proposes the development of low-power hardware based on power converters to carry out experimental tests. The basic architecture of the microgrid is formed by two power ports driven by their respective DC/DC converter, which are connected through a DC bus where DC loads or additional sources can be connected. An off-grid inverter is also available to feed the AC loads as shown in Fig. 1. The system also has a communication network to transmit the measured variables, which are sent from two microcontrollers, called Slave 1 and Slave 2, to a third microcontroller, called Master. The Master device interacts with a PC through USB serial communication for monitoring and management. For Fig. 1 the dotted lines represent the signal communication network, and the solid line represents power and measured signals.

### 2.1 DC bus description

The DC bus is the main connection node of the microgrid; therefore, for simple connection of the hardware elements, a pair of copper bars are the physical DC bus voltage; for this work, the DC bus voltage is 36 V, but 48 V can be selected as well. DC bus bars can support 30A. These bus bars allow the interconnection of DC/DC converters, DC loads, inverter input, and other DC equipment, ensuring a secure connection and low resistance to minimize power losses in the system.

**Fig. 2:**
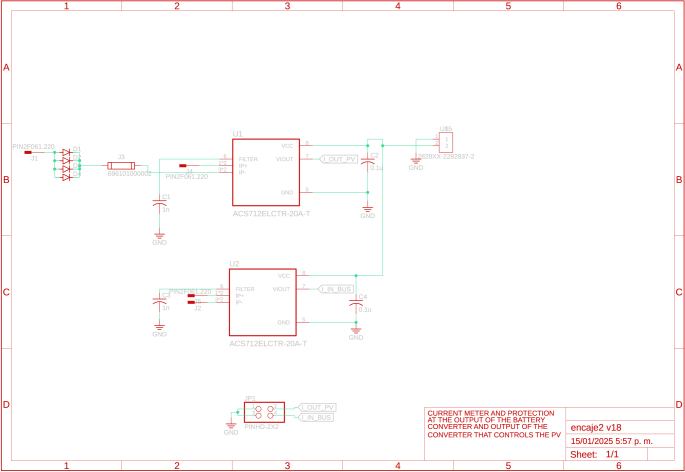
DC bus current sensor board.

An electronic board was developed to measure the input and output currents on the DC bus, i.e., the currents delivered by the sources and their converters or consumed by the loads and their converters. The current measurement uses the hall effect sensor ACS712-30, which allows measuring currents in a range of −30A to 30A. This board requires a supply voltage of 5 V for proper operation; the schematic of the measurement board is presented in Fig. 2.

### 2.2 Power modules and instrumentation

**Fig. 3:**
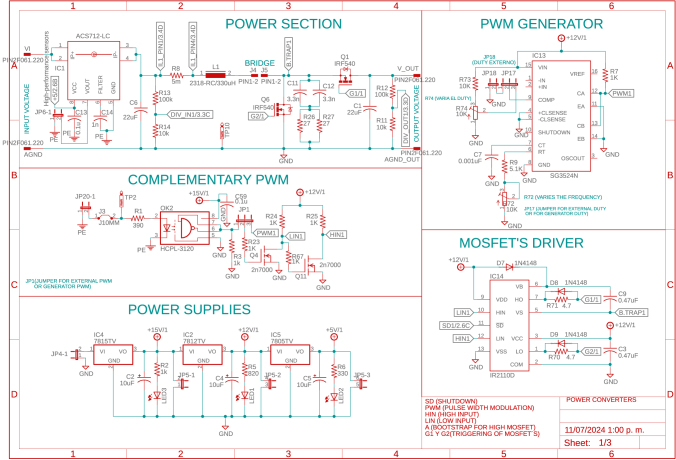
First power converter module.

**Fig. 4:**
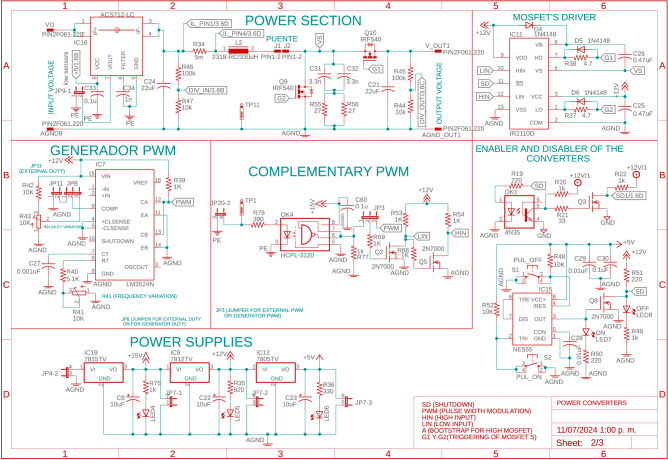
Second power converter module.

**Fig. 5:**
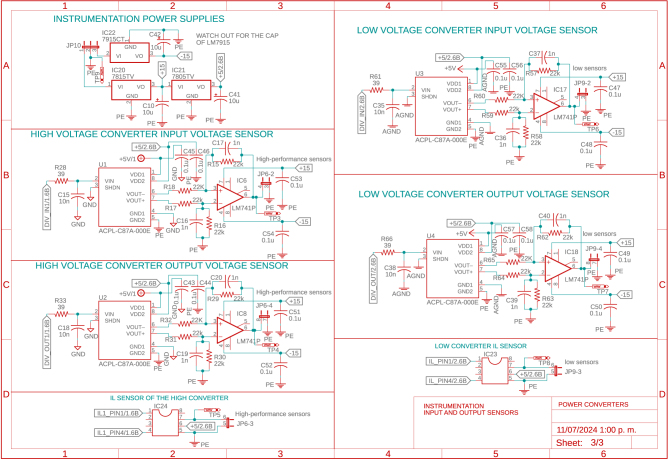
Instrumentation for power converter modules.

This stage has two power input and output ports, each managed by a bi-directional boost converter. Each power input can operate from 5 V to 100 V and a maximum of 5.2A. IRF3710 MOSFETs, 22μF capacitors for input and output, and 330μH inductors were used to construct the Boost converters, inductors, and capacitors were calculated to operate within the defined range and maintain low voltage and current ripple. Each power input is equipped with instrumentation circuitry, including voltage regulators, drivers, and other essential components to ensure proper operation; the power and instrumentation modules are presented in Fig. 3, Fig. 4, Fig. 5.

**Fig. 6:**
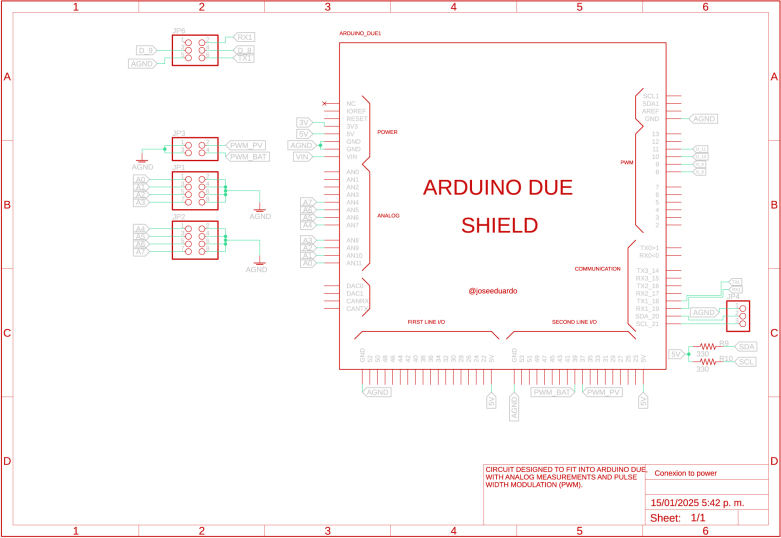
Coupling board for Slave 1 and power converter modules.

The power circuitry also features an enable circuit for each converter, allowing all converters to be turned on and off simultaneously via the 555 integrated circuit (IC). This design enables the board to operate safely in several renewable energy applications because it allows the proper connections and disconnections before turning on/off the power modules. In addition, each power circuit on the board features voltage and current measurements on capacitors and inductors, which is achieved with ACS712 optocoupled sensors and the ACPL-C87A-00E, ensuring accurate and safe measurements. The converters’ signals are captured and sent to an Arduino DUE microcontroller (Slave 1). In this same device, the controllers for each converter are implemented. For the connection between the power converter board and the microcontroller, the coupling board shown in Fig. 6 is used, which uses a header connector that connects the signals measured on the power board and the control signals (PWM’s) from the terminals of the Slave 1.

### 2.3 DC/DC buck converter and power inverter for AC loads

A commercial modified waveform inverter, rated at 1000W, powers the AC loads. The inverter’s input voltage is 12 V, providing an output of 100V—240V RMS, with an efficiency of 90% and a frequency of 60Hz. This inverter is suitable for many household and electronic loads but is not ideal for sensitive equipment, high-power motors, or medical equipment.

**Fig. 7:**
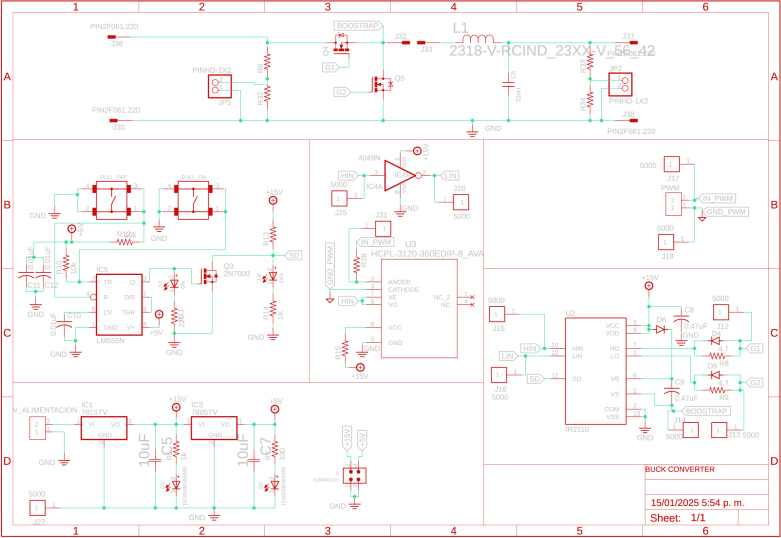
Buck converter and instrumentation.

A Buck converter allows the coupling of the inverter with the DC bus, the converter shown in Fig. 7 adjusts its output voltage to the level required for the inverter. Additionally, this implementation allows measuring the power delivered to the inverter and disconnecting it from the DC bus when desired. With an inverter that handles a voltage range similar to that of the DC bus voltage, the buck converter is not required for the coupling, simplifying the system. This design ensures that power needs can be met efficiently and safely despite the limitations of modified wave inverters regarding compatibility with sensitive equipment.

**Fig. 8:**
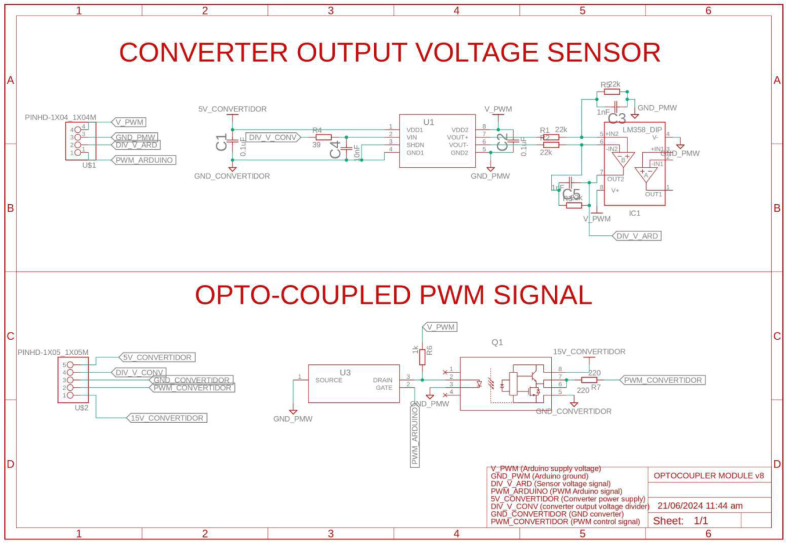
Sensing and optocoupling electronic board.

Additionally, a electronic board that allows optocoupling voltage signals for measurement and control of the converter is implemented. Therefore, the differential voltage sensor ACNT-H87B-000E reads the voltage at the output of the Buck converter. On the other hand, the HCPL-3120 optocoupler is used to isolate the control signal coming from Slave 2. Fig. 8 shows the previously described electronic board with its respective conditioning circuits.

Slave 2 measures DC voltage $v_{dc_{link}}$ and current $i_{dc_{link}}$ at the connection point located at the output of the buck converter and input of the inverter. Also, Slave 2 performs a control for the voltage at the output of the buck converter to ensure proper operation and maintain the integrity of the inverter due to its working specifications; in this sense, the variable $v_{dc_{link}}$ helps to regulate the voltage using a PI controller. In addition, the variables $i_{dc_{link}}$ and $v_{dc_{link}}$ flow to the Master through the communication system to determine the power consumption by the AC loads connected to the inverter.

### 2.4 Communication system

In order to obtain power measurements from the test bench and analyze its performance, a communication system has been implemented to collect data from each of the elements connected to the microgrid. This system uses a half-duplex master–slave communication architecture. Measurements and communication interfaces allow continuous monitoring of the power generated and consumed as well as the current and voltages of the microgrid. The communication system requires a master device to request and receive data from the Slave devices. With the information collected, the behavior of the different elements connected to the microgrid is graphically presented. Additionally, the Master sends the collected data to a central processor that can visualize, process, and make decisions based on the collected information.

The data acquisition system uses the RS485 protocol, operating at 115200 bps and readily accommodating additional devices. Its differential signaling, which requires only two communication lines and supports cable lengths up to 1200 m, makes it widely used in industrial applications.

**Fig. 9:**
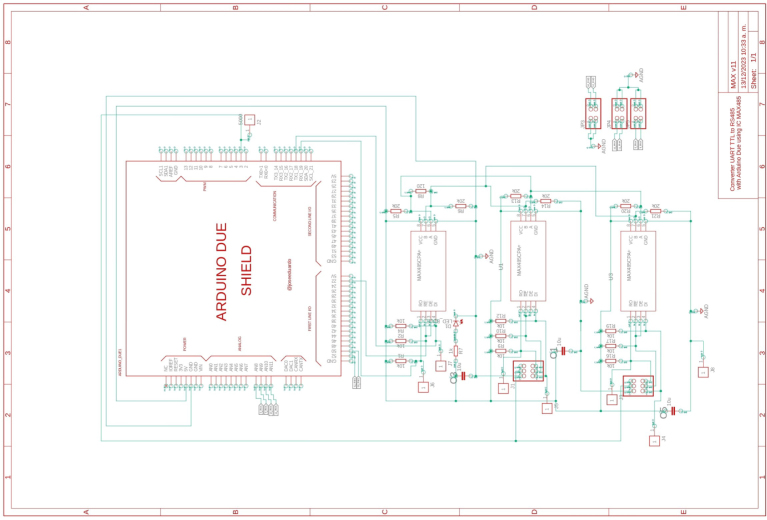
Communication system.

To implement this topology with the microcontroller Arduino DUE, it is necessary to use the IC MAX485, which converts UART data to RS485. The schematic of the ICs and their respective conditioning circuits are shown in Fig. 9. Additionally, since UART communication is asynchronous, a protocol was designed to establish synchronous communication from the Master to each of the slaves. This procedure was implemented at both hardware and software levels using an enabling pin that allows changing the mode from transmit to receive. The two Slave devices were programmed using the Arduino IDE software, while the Master was programmed using Simulink.

### 2.5 Power management system

The Power Management System (PMS) is designed to provide a graphical interface that facilitates the observation and recording of power generation and consumption variables. In the Fig. 1 it can be seen that Slave 1 takes the power parameters from the converters connected to power ports PP1 and PP2. Slave 2 collects power data from the inverter and DC loads. According to the above, the data collected and observed under the PMS are PP1 voltage ($v_{pp1}$), PP1 current ($i_{pp1}$), PP2 voltage ($v_{pp2}$), PP2 current ($i_{pp2}$), DC link voltage $\left(v_{dc_{link}}\right)$, DC link current $\left(i_{dc_{link}}\right)$, and current of the loads $\text{(}i_{load1}$, $i_{load2}\text{)}$.

**Fig. 10:**
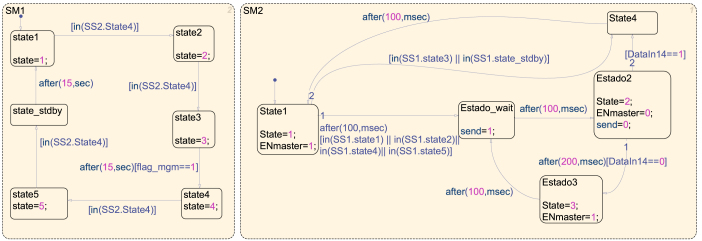
Finite State Machine.

Fig. 10 illustrates the system’s communication process using two finite-state machines (FSM). The first state machine (SM1) manages the master’s data reception and transmission. The second state machine (SM2) defines the sequence of information exchange between the master and the slave devices.

The SM1 state machine consists of five main states and a standby state. Its behavior is described as follows:

• **State**$S_{1}$:
  – Enables communication from slave 1 → master.
• **State**$S_{2}$:
  – Enables communication from slave 2 → master.
• **State**$S_{3}$:
  – Once data from both slaves has been received, the management algorithm is executed.
  – The transition between $S_{1}\to S_{2}$ and $S_{2}\to S_{3}$ occurs when the SM2 state machine has completed a cycle and entered state $S_{4}$.
• **Transition**$S_{3}\to S_{4}$:
  – Before transitioning to state $S_{4}$, the system must compute the reference values and activate the flag_management signal, which indicates that the management algorithm has been completed.
  – The transition occurs when at least 15 s have elapsed and the flag is set.
• **States**$S_{4}$**and**$S_{5}$:
  – $S_{4}$: Enables communication from master → slave 1.
  – $S_{5}$: Enables communication from master → slave 2.
  – Once both transmissions have been completed, the system enters a standby state for 15 s before restarting at state $S_{1}$. During this period, the system operates using the reference values provided by the management system.

The SM2 state machine consists of three main states and three standby states. Its operation is described as follows:

• **State**$S_{1}$:
  – Requests data from the master to the selected slave.
  – If the SM1 state machine is in state $S_{3}$ (management algorithm) or in standby, a transition to state $S_{4}$ occurs, where the system waits for 100 ms.
  – Otherwise, if SM1 is in states $S_{1},S_{2},S_{4}$, or $S_{5}$, the system enters a waiting state for 100 ms before transitioning to the next state, $S_{2}$.
• **State**$S_{2}$:
  – Handles the reception of data from the slave devices.
  – If no response is received after 200 ms, the system transitions to state $S_{3}$, where the master reattempts data requests to the slaves.
  – Subsequently, the system enters another waiting state of 100 ms before proceeding.
• **Transition**$S_{2}\to S_{4}$:
  – Once the responses from the slaves are received, the system transitions from state $S_{2}$ to state $S_{4}$.
  – After 100 ms, the system returns to state $S_{1}$ to restart the cycle.

**Fig. 11:**
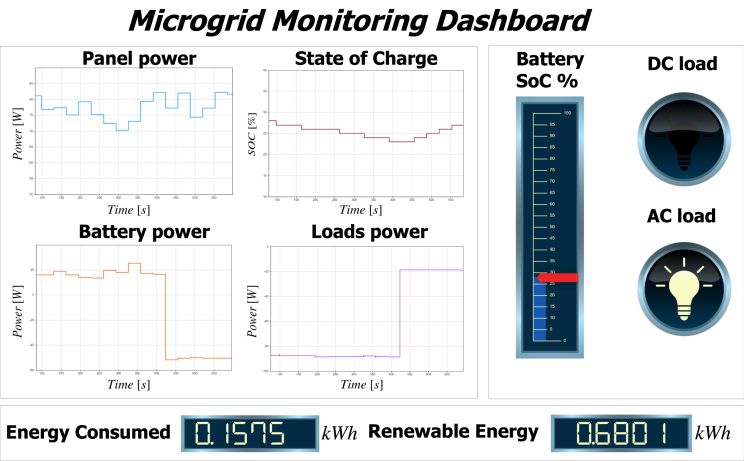
Dashboard interface.

Fig. 11 illustrates the platform’s monitoring interface for power generation and consumption. The upper left section displays real-time graphs of key parameters acquired by the Master, including panel power, battery power, battery SOC behavior, and power consumption of AC and DC loads. The upper right section indicates the current battery charge percentage. Additionally, the interface presents the loads’ current on/off status, which the PMS controls. Finally, the lower section provides data on energy consumption and generation. The Software/Master directory of the online repository contains the Simulink block code.

## 3 Design files summary

The design files are available in the online repository [20] (see Table 2).

**Table 2:** Summary of all design files.

Desing file name	File type	Open source license	Location of the file
Master(.brd)(.sch)	Fusion360, Fig. 9 PCB desing	CERN-OHL-P	Hardware/Master
SL1PCCoupler(.brd)(.sch)	Fusion360, Fig. 6 PCB desing	CERN-OHL-P	Hardware/SL1PCCoupler
Instrumentation1(.brd)(.sch)	Fusion360, Fig. 2 PCB desing	CERN-OHL-P	Hardware/Instrumentation1
BuckConverter(.brd)(.sch)	Fusion360, Fig. 7 PCB desing	CERN-OHL-P	Hardware/BuckConverter
PowerConverters(.brd)(.sch)	Fusion360, Fig. 3, 4, y 5 PCB desing	CERN-OHL-P	Hardware/PowerConverters
Instrumentation2(.brd)(.sch)	Fusion360, Fig. 8	CERN-OHL-P	Hardware/Instrumentation2
Base assembly(.f3d)	Fusion360, Fig. 12(a) 3D design	CERN-OHL-P	Hardware/Case Assembly
Front side assembly(.f3z)	Fusion360, Fig. 12(b) 3D design	CERN-OHL-P	Hardware/Case Assembly
Rear side assembly(.f3z)	Fusion360, Fig. 13(a) 3D design	CERN-OHL-P	Hardware/Case Assembly
Left side assembly(.f3d)	Fusion360, Fig. 13(b) 3D design	CERN-OHL-P	Hardware/Case Assembly
Right side assembly(.f3d)	Fusion360, Fig. 14(a) 3D design	CERN-OHL-P	Hardware/Case Assembly
Shell side assembly(.f3z)	Fusion360, Fig. 14(b) 3D design	CERN-OHL-P	Hardware/Case Assembly
Intermediate platform assembly(.f3d)	Fusion360, Fig. 15(a) 3D design	CERN-OHL-P	Hardware/Case Assembly
Intermediate platform installing(.f3d)	Fusion360, Fig. 15(b) 3D design	CERN-OHL-P	Hardware/Case Assembly

## 4 Bill of materials summary

The BOM of the PCBs implemented in the microgrid can be found in the repository. The files are presented in the Section 3 (see Table 3).

**Table 3:** List of BOM and prices.

Module	File type	Cost (USD)	Location of the file
BOM_Master.xlsx	Fig. 9 BOM	78.81	Hardware/Master
BOM_Instrumentation1.xlsx	Fig. 2 BOM	13.35	Hardware/Instrumentation1
BOM_Instrumentation2.xlsx	Fig. 8 BOM	17.41	Hardware/Instrumentation2
BOM_BuckConverter.xlsx	Fig. 7 BOM	82.43	Hardware/BuckConverter
BOM_PowerConverters.xlsx	Fig. 3 BOM	121.75	Hardware/PowerConverters
BOM_SL1PCCoupler.xlsx	Fig. 6 BOM	49.11	Hardware/SL1PCCoupler
BOM_HardwareCase.xlsx	Fig. 17(a) BOM	53.27	Hardware/Case Assembly

	**TOTAL**	416.13	

## 5 Build instructions

**Fig. 12:**
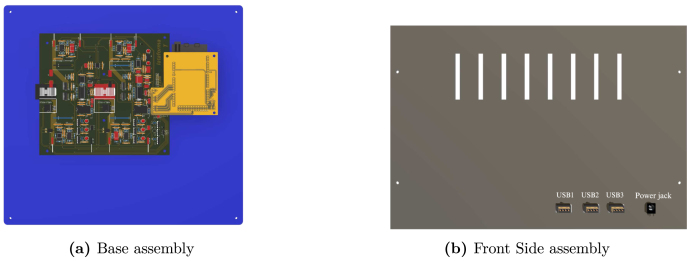
Base and Front side assembly.

**Fig. 13:**
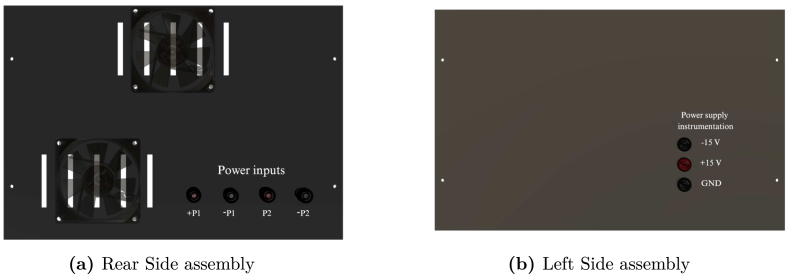
Rear Side and Left Side assembly.

**Fig. 14:**
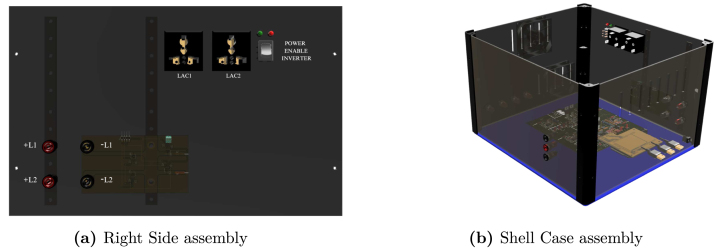
Right Side assembly and Shell Case assembly.

**Fig. 15:**
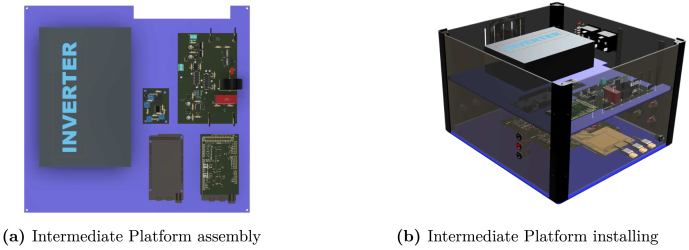
Intermediate platform assembly and installing.

For the hardware construction, it is necessary to build the PCBs presented in Fig. 3, Fig. 4, Fig. 4 from the Gerber files, the designs are presented in schematic (.sch) and board (.brd) files. Any PCB design software can import, edit, and update these files if required. Additionally, the hardware case parts presented in [20] must be cut out. Once these elements are available, proceed to assemble the hardware according to the following steps:

• **Step 1. Base assembly**: Using the SL1PCCoupler board, attach the Slave 1 and the Power Converter circuit. On the baseboard (Fig. 3), fit the circuits using threaded standoff and screws, preferably plastic, as shown in the Fig. 12(a).
• **Step 2. Front Side assembly**: On the Front Side (Fig. 12(b)), install the USB ports for programming the Arduino and the jack for the power supply.
• **Step 3. Rear Side assembly**: Install the terminals for power inputs 1 and 2 and the two fans on the Rear Side as shown in the Fig. 13(a).
• **Step 4. Left Side assembly**: On the Left Side, for the power supply of the Instrumentation and Sensor electronic board belonging to the Power Converter circuit, connect three jack terminals and one screw terminal block as shown in Fig. 13(b).
• **Step 5. Right Side assembly**: On the inner side of the Right Side, install the DC bus bar vertically using two jack terminal blocks. With another two jack terminals, install the Instrumentation1 circuit internally connected to the negative DC bus bar. Install the inverter output connectors, the on/off switch, and the LED indicator on this side. Fig. 14(a) shows the final result of the assembly.
• **Step 6. Shell Case assembly**: Adapt the CornerSupports on the Base and install the Rear, Left, and Right Sides. Using the corresponding wires, connect all the instrumentation power input and power supply terminals; connect the positive power outputs of the Power Converter circuit to the positive DC bus bar and the negative outputs to the Instrumentation1 circuit. Fig. 14(b) shows the assembled case.
• **Step 7. Intermediate platform assembly**: On the intermediate platform, install the inverter, the BuckConverter, the Instrumentation2 circuit, the Slave 2, and Master microcontrollers as shown in the Fig. 15(a). Install the intermediate platform on top of the Shell Case as shown in Fig. 15(b).

**Fig. 16:**
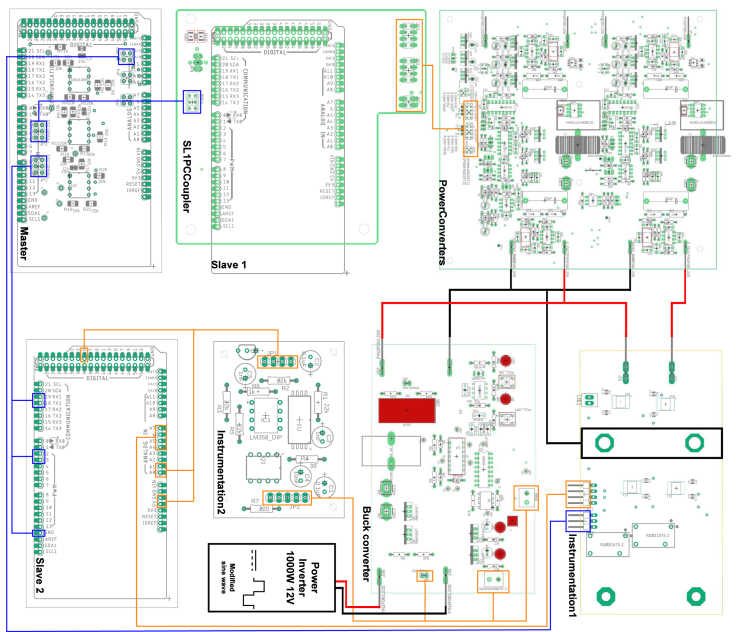
Electronic boards connections.

The connection of the boards is shown in Fig. 16. The red and black lines indicate the connections corresponding to the power stage. The orange lines indicate the data acquired by the Instrumentation1 and Instrumentation2 electronic boards and transmitted to Slave 1 and Slave 2. Finally, the blue lines represent the connections of the communication system between the Master and the two Slaves.

**Fig. 17:**
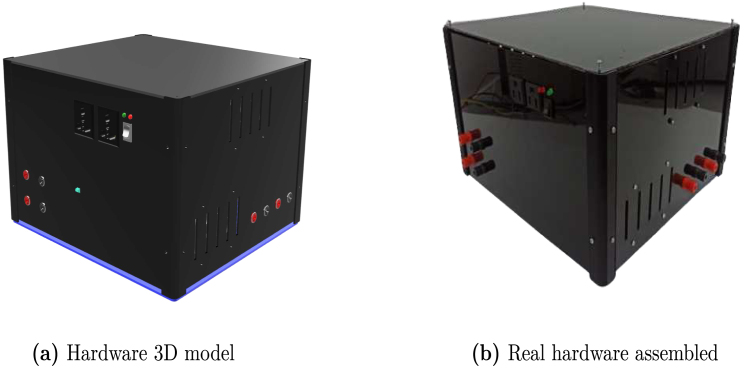
Hardware 3D model and real hardware assembled.

Once the boards and devices are connected, place the box’s top cover, finalizing the hardware assembly as shown in Figs. Fig. 17, Fig. 17 for the 3D model and the actual hardware assembled in detail.

The platform supports various input and output connections with varying voltage and current levels, as detailed in Table 4. These connections provide AC load outputs, instrumentation power supplies for the power modules—maintained at +15V, −15V, and GND by the employed regulators—power supply interfaces for storage or generation systems, and USB ports for data transfer to various microcontrollers along with their dedicated power supplies. This architecture accommodates a wide range of energy sources, ensuring compatibility with diverse loads and power components, while the specified limits help prevent system overloads and maintain operational stability.

**Table 4:** Table of input/output ports and electrical characteristics.

Ports	Direction	Symbol	Voltage level	Current level
Power supply instrumentation	IN	PSI	+15, −15 V GND	0.5 A
USB	IN	USB 1,2,3	5 V	0.5 A
Power jack	IN	PJ	5 V	0.5 A
Power DC input 1	IN/OUT^b^, ^c^	+P1, −P1	15–25 V	5 A
Power DC input 2	IN/OUT^b^, ^c^	+P2, −P2	15–25 V	5 A
Load DC 1	OUT/IN^a^, ^b^	L1+, L1−	36–40 V	5 A
Load DC 2	OUT/IN^a^, ^b^	L2+, L2−	36–40 V	5 A
Load AC 1	OUT^b^	LAC1	100–240 RMS	8 A
Load AC 2	OUT^b^	LAC2	100–240 RMS	8 A

a Allows connection of load and bidirectional sources.

b Manipulable port.

c Allows connection of bidirectional sources.

## 6 Operation instructions

The equipment composing the microgrid hardware plays a fundamental role in its operation, so it is essential to establish operating instructions that allow the microgrid to be fully functional. First, it is crucial to have a controller designed for the power converters on the platform, this ensures a stable operation of the system and the management of the parameters of the microgrid for the desired operating specifications. Secondly, it is essential to properly turn on and off the equipment that composes the microgrid to avoid the instrumentation and control systems. The following is a step-by-step guide to putting the microgrid into correct and safe operation.

**Fig. 18:**
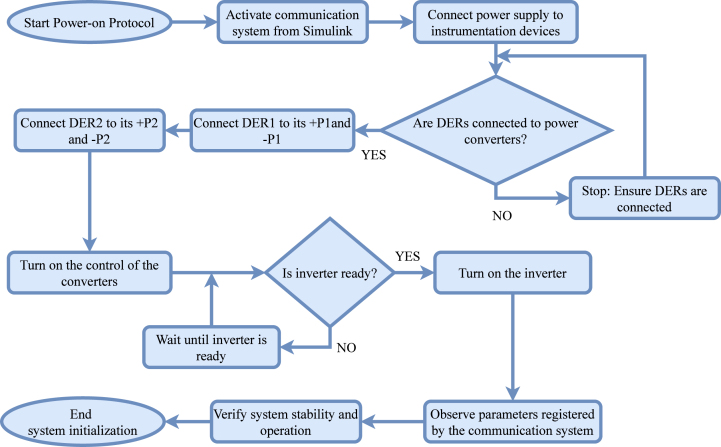
Power-on protocol.

A proper power-on and power-off procedure ensures that the power and communication system operates safely. Fig. 18 illustrates the correct power-on sequence, which users must follow to ensure all components receive power and function correctly.

To initiate the startup process, users must first activate the communication system, which initializes the Simulink interface for data transfer and system monitoring. Next, they power the instrumentation devices to ensure accurate data acquisition. Once the instrumentation operates correctly, they connect the system to the power generation ports, which distribute electrical power and enable controlled energy integration. Activating the power module platform ensures the proper functioning of the system’s power modules. Subsequently, turning on the power inverter converts DC to AC, allowing load integration. Finally, users connect and supply power to the AC and DC loads. Once all components receive power, they must review the recorded parameters in the communication system to verify system stability and proper operation before proceeding with further tasks.

**Fig. 19:**
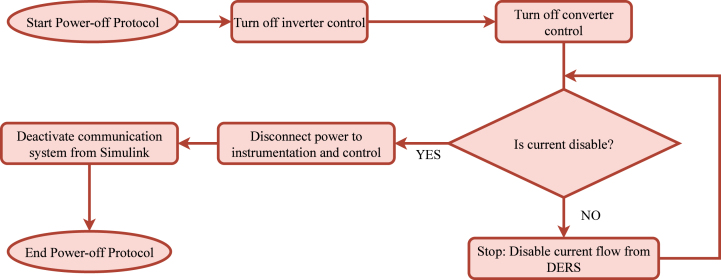
Power-off protocol.

A specific sequence must be followed to safely shut down the system, as illustrated in Fig. 19. The shutdown process begins by turning off the power inverter and deactivating the power module platform to prevent further energy generation or extraction, effectively interrupting the power flow from the DER. Next, the power supply to the instrumentation ports is disconnected to ensure that measurement devices are safely powered down. Finally, the communication system is deactivated to complete the shutdown process. Before concluding, verifying that the current flow is completely interrupted is essential to confirm that the system has been safely powered off. This protocol ensures that the system operates safely during the power-up and shutdown phases, preventing damage to components and enhancing overall performance.

## 7 Validation and characterization

**Fig. 20:**
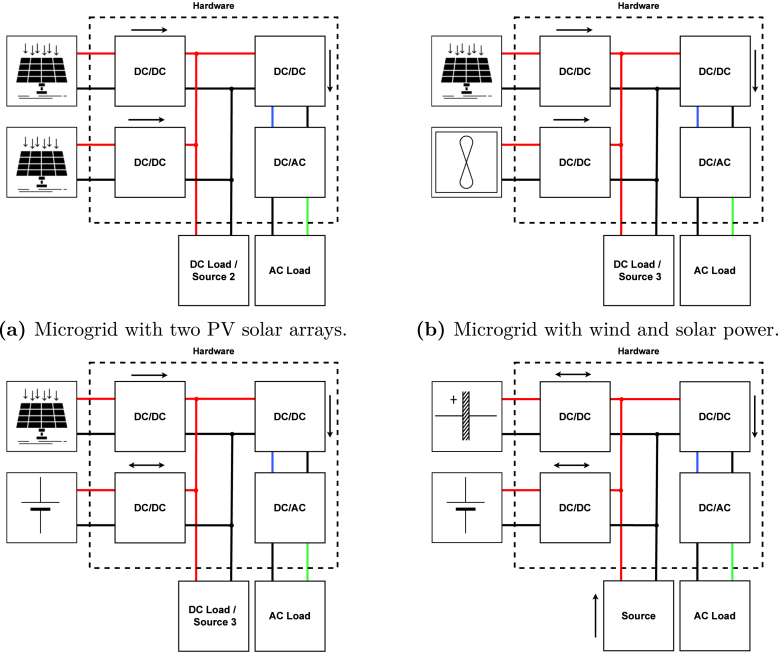
Test scenario alternatives.

The presented hardware allows exploring different test scenario alternatives, such as those presented in the Fig. 20. The first scenario (see Fig. 20(a)) proposes the use of two solar panels or arrays as distributed generation sources, through which distributed maximum power point tracking (DMPPT) strategies can be explored. The second scenario (see Fig. 20(b)) proposes to include another type of source, such as a wind generator with rectifier, through which it is possible to test Maximum Power Point Tracking (MPPT) techniques and obtain generation conditions dependent on different variable than solar radiation. The third scenario proposes using an energy storage system such as a battery, by which it is possible to have an energy backup for periods in which the photovoltaic generation is lower than the demand of the loads. In this case, the battery converter must operate bi-directionally to allow charging and discharging of the battery (see Fig. 20(c)). Finally, the fourth scenario proposes using a hybrid battery-ultracapacitor storage system, for which it is possible to connect on the DC bus another controlled power source that charges the storage elements as seen in Fig. 20(d).

**Fig. 21:**
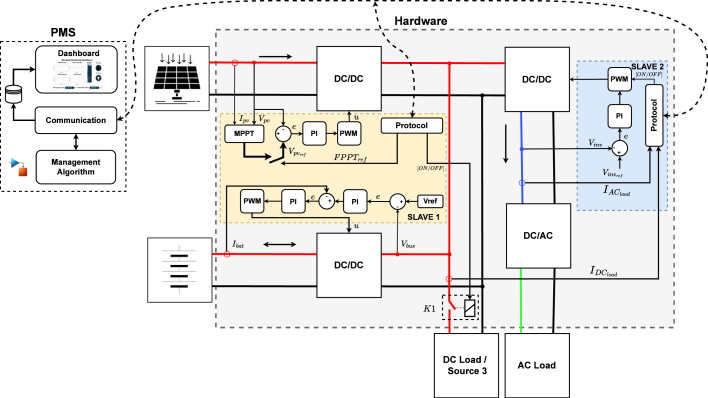
Scenery for testing the proposed hardware.

The third scenario was selected to validate the hardware performance, as shown in more detail in the sample control strategies for each source and load in Fig. 21. The figure illustrates the structure of the controllers, which are programmed in Slave 1 and Slave 2. These slave devices communicate with the master, transmitting the measured variables and receiving control actions and reference values. The reference values may vary depending on the programmed strategy. The PMS processes the data received from the slaves. If an active management strategy is in place, the PMS executes it, displays the data on the dashboard, and sends the reference values defined by the management algorithm. Otherwise, the system displays the received variables on the dashboard.

For the PV panel, a PI control regulates the PV voltage to the value defined by the Perturb and Observe (P&O) MPPT algorithm. The MPPT algorithm requires measuring the PV current and PV voltage to calculate the voltage reference for the voltage controller. For the voltage control, the parameters are $K_{p}=-0.00656\;1/V$, $K_{i}=-0.00969\;1/V\cdot s$ and the sampling time $t_{s}=0.1\;\mathrm{ms}$; and for the P&O algorithm, the perturbation voltage is ΔV=0.5V and the perturbation time is $t_{p}=2\;\mathrm{ms}$.

For the battery, a cascade control loop regulates the bus voltage at $v_{bus}=36\;V$. The measurements required for this control are battery current and bus voltage. The parameters for the internal current control are $K_{p}=0.00049\;1/A$, $K_{i}=0.08724\;1/A\cdot s$ and the sampling time $t_{s}=2\;\mathrm{ms}$; while for the voltage control the parameters are $K_{p}=0.085\;A/V$, $K_{i}=0.00066\;A/V\cdot s$ and $t_{s}=10\;\mathrm{ms}$.

A buck converter is responsible for supplying power to the inverter; it reduces the 36 V from the DC bus to 12 V for the input of the inverter; also, a PI voltage control regulates the voltage at the inverter input. The inverter feeds the AC loads with a $120\;V_{RMS}$. The parameters of the voltage controller for the buck converter are $K_{p}=0.745\;1/V$, $K_{i}=0.00619\;1/V\cdot s$ and $t_{s}=1\;\mathrm{ms}$.

Slave 1 runs the PV panel and battery controllers, Slave 2 runs the voltage control for the inverter input. All measurement data are sent via the communication protocol to the Master and displayed on the Dashboard.

### 7.1 Testbench description

**Fig. 22:**
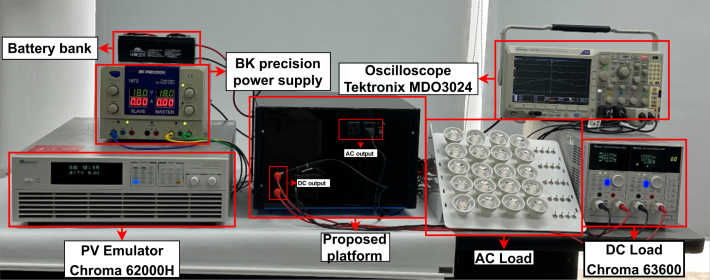
Testbench for the proposed hardware testing.

For validation experiments, the testbench construction requires power sources, storage elements, and loads shown in Fig. 22 and detailed next:

• **PV Emulator Chroma 62000H** DC voltage source to emulates power and current curves for PV panels at several irradiance and temperature conditions. The software Solar Array allows programming the DC source.
• **Battery bank** The battery bank uses a series of two batteries FL1223GS with 12 V of nominal voltage and 2.3Ah of nominal capacity as the storage element connected to the bidirectional power converter.
• **DC Load Chroma 63600** Programmable DC load with 500mA required current.
• **AC Load** No-linear load using twenty LED bulbs of 7W.

In addition to the above devices, a power supply feeds the instrumentation and control devices for the power converters. An oscilloscope measures the testbench’s different current and voltage waveforms. The PV Emulator is configured with a standard irradiance of $1000\;{\mathrm{W/m}}^{2}$, a temperature of 25°C, a maximum power of 50W, a fill factor of 0.75 and a voltage temperature coefficient of −0.38%. To test the hardware in different operating conditions, the two scenarios below allow the validation when the PV source does not supply power, and the batteries feed the loads; on the contrary, the PV panel supplies power to loads, and excess goes to the batteries.

### 7.2 Test case 1: Stability under low PV generation and load transients

The PV generator is set to deliver 4% of the maximum power for the first validation test. The battery, its converter, and controller maintain the bus voltage at 36 V and supply the power required by the loads. The AC and DC loads are turned on and off to observe the microgrid’s stability and behavior against changes in power consumption.

**Fig. 23:**
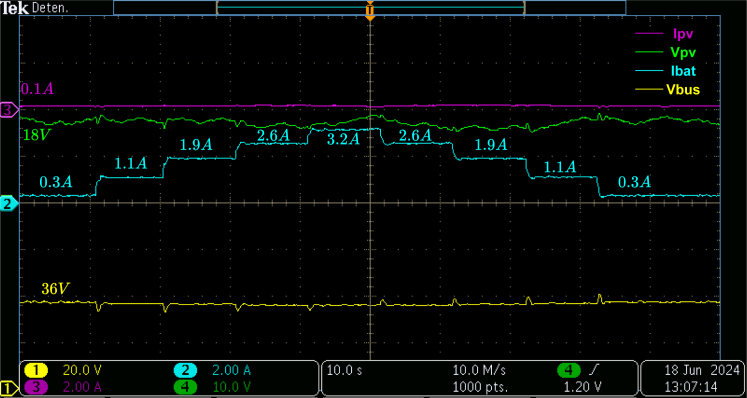
Low PV power and batteries feeding changing loads.

Fig. 23 shows the PV panel voltage and current, the battery current, and the DC bus voltage; the PV generator delivers approximately 1.8W, which is lower than the power demanded by the loads, so the energy storage system must supply the remaining power, thus changes in the current value are observed between a range of 0.3A and 3.2A, which corresponds to a range of power delivered from the battery between 7.2W and 76.8W. In addition, the stability in the DC bus voltage against the changes generated by the connection and disconnection of loads allows verifying that the control and the converter connected to the battery guarantee a stable operation against possible disturbances.

### 7.3 Test case 2: Dynamic PV generation reduction and battery response under sequential load variations

**Fig. 24:**
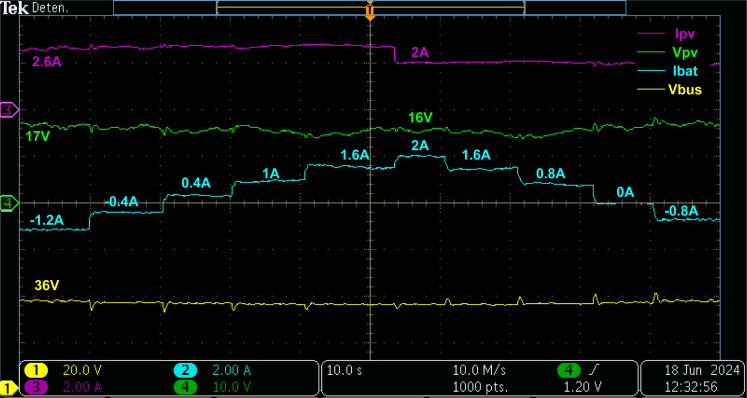
Changes on PV power and the loads.

For the second validation test, the PV generator supplies 90% of peak power and changes to 75% at 5.2s. Again, the loads turn on and off sequentially to observe the behavior of the energy storage system. Fig. 24 show the PV voltage and current, the battery current, and the DC bus voltage parameters. The figure shows that the PV panel generates approximately 44.2W; then, the generation is substantially reduced to 32W and remains around that value. In both scenarios, the MPPT control is active, which allows the converter to deliver the maximum power value generated by the PV panel.

On the other hand, the battery current is initially negative since the power generation exceeds the demand; therefore, the battery starts to charge. However, as the loads increase, the battery discharges to supply the missing power. In addition, the battery current increases as the power generation decreases, demonstrating the microgrid’s stability. Finally, the DC bus voltage is stable despite disturbances presented in both power demand and generation, which reaffirms the microgrid’s control and stability.

### 7.4 Test case 3:

Power Management Strategy - Hierarchical control

This subsection presents the energy management strategy implemented in the proposed platform, which allows for managing the generation and consumption of energy between renewable sources, storage systems, and connected loads. This hierarchical strategy guarantees the system’s stable operation, prioritizing the use of renewable energy and managing the SOC of the batteries to maximize the autonomy and stability of the microgrid.

The management algorithm balances generation and demand while considering the system’s operating conditions. This approach enables the system to decide when to activate the MPPT and FPPT (Fixed Point Power Tracking) algorithm, control the energy flow to or from the batteries, and manage critical loads by disconnecting them when necessary.

**Fig. 25:**
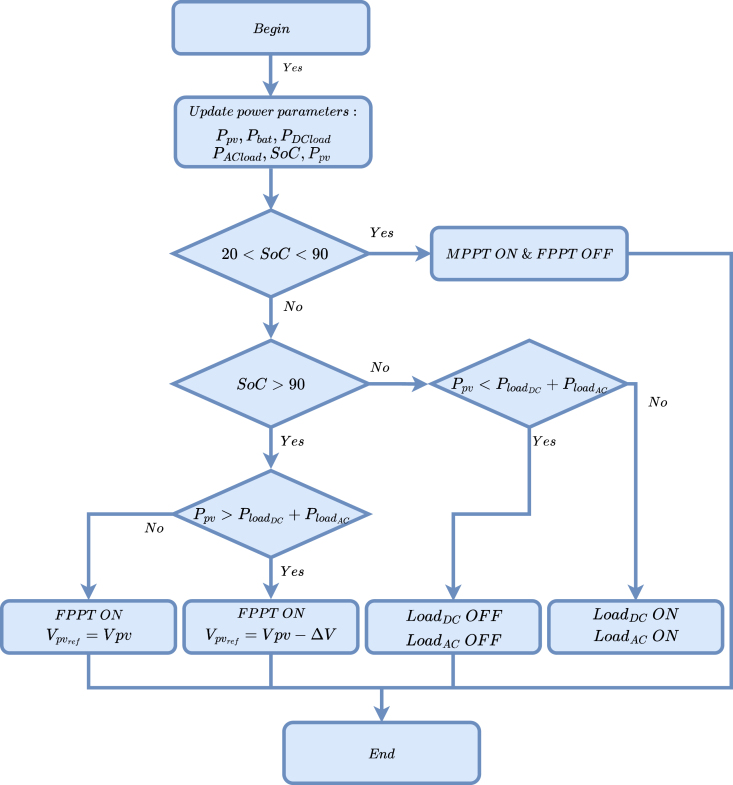
Flowchart of the energy management strategy.

Fig. 25 illustrates the flow chart of the energy management strategy implemented in the platform. This diagram represents the hierarchical control logic balancing energy generation, storage, and consumption. The process starts with updating key energy parameters, including PV power $P_{pv}$, battery power $P_{bat}$, DC and AC load powers $P_{DC_{load}},P_{AC_{load}}$, battery SOC and PV system generated power $P_{pv}$. The first decision node evaluates whether the SoC is within the operating range (20% ≤ SoC ≤ 90%). If so, the MPPT algorithm is enabled, and FPPT is disabled, maximizing solar energy harvesting. If SoC exceeds 90%, control focuses on distributing the generated energy. The system compares the available PV power with the total load demand (DC and AC). If Ppv is higher than the total demand, FPPT is activated with a reduced operating point $V_{pv_{ref}}=V_{pv}-\Delta V$ to avoid battery overcharging. Otherwise, if $P_{pv}$ cannot cover demand, the system can perform load shedding by turning off DC and AC loads to maintain operational stability.

**Fig. 26:**
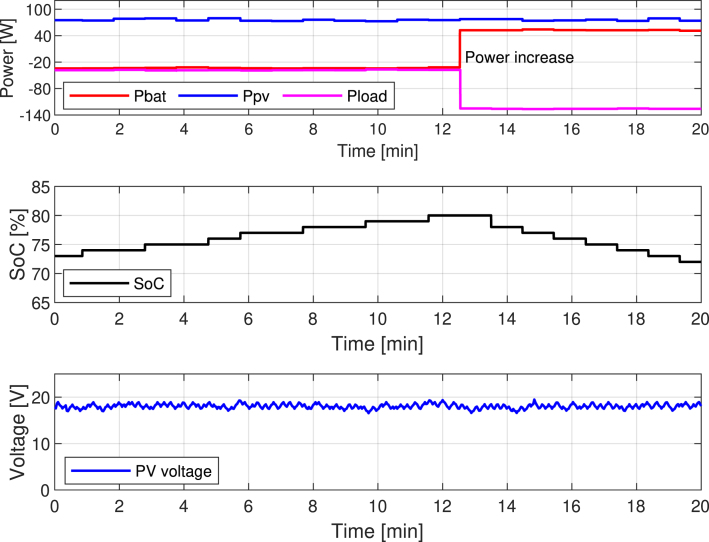
Experimental validation of the energy management algorithm during dynamic load conditions.

Fig. 26 displays the parameters that the graphical interface collects, including $P_{pv}$, $P_{bat}$, and the power consumed by the AC and DC loads. The system implements a load profile to analyze battery performance. The AC and DC loads in this profile initially consume approximately 40 W. At minute 13, power consumption significantly increases to 120 W. In response, the battery adjusts its behavior, supplies the required power, and discharges.

The middle plot of Fig. 26 reflects this dynamic by showing the battery’s SOC. Initially, the panel generates surplus energy, which charges the battery and gradually increases the SOC from approximately 72% to 81%. When power demand rises, the battery discharges to compensate for the additional load. As a result, the SOC steadily declines after minute 13, reaching about 73%.

The bottom plot displays the PV panel voltage throughout the experiment. It remains relatively stable around 18–20 V, indicating that the MPPT algorithm maintains the panel near its optimal operating point despite fluctuations in power demand. This stability ensures efficient energy harvesting from the PV source, even during dynamic load conditions.

**Fig. 27:**
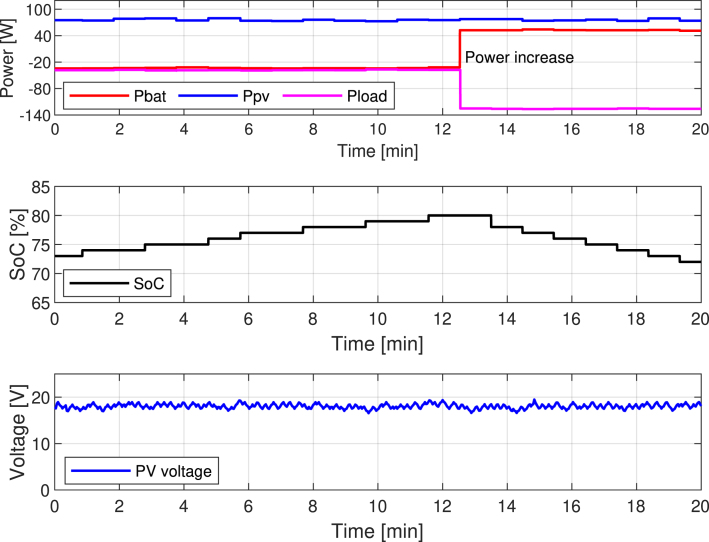
Experimental validation of the MPPT to FPPT transition.

Fig. 27 presents a new scenario where the system starts with an initial SoC of 85%. The PV generator delivers its peak power, approximately 80 W, while the system maintains a constant consumption profile of about 55 W. The PMS allows battery charge and discharge within the 20% to 90% range.

As shown in Fig. 27, the battery is initially charging due to the surplus energy from the PV generator. Once the SoC reaches the upper allowed limit (90%), the PMS instructs Slave 1 to deactivate the MPPT algorithm and switch to FPPT. To execute this transition, the Master controller sends a voltage reference to Slave 1, which then adjusts the PV panel voltage to reduce power generation progressively. This process ensures that the battery is not overcharged and prevents energy wastage.

The top plot in Fig. 27 illustrates this transition, where the Ppv (blue line) decreases gradually after minute 13, corresponding to the activation of the FPPT. Meanwhile, the battery power (Pbat, red line) also decreases, indicating reduced charging current, eventually stabilizing around 0 W as the PV output aligns with the load demand.

The middle plot displays the SoC evolution, where the battery continues to charge even after reaching 90%, peaking at 92% due to the gradual nature of the FPPT regulation. This slight overcharge highlights the smooth control process, which avoids abrupt transitions that could destabilize the system.

The bottom plot shows the PV panel voltage behavior. Initially, the voltage remains around the maximum power point under MPPT control. After the switch to FPPT, the voltage increases as the system reduces the PV output, eventually settling at a higher voltage level that limits power generation to match the load demand.

**Fig. 28:**
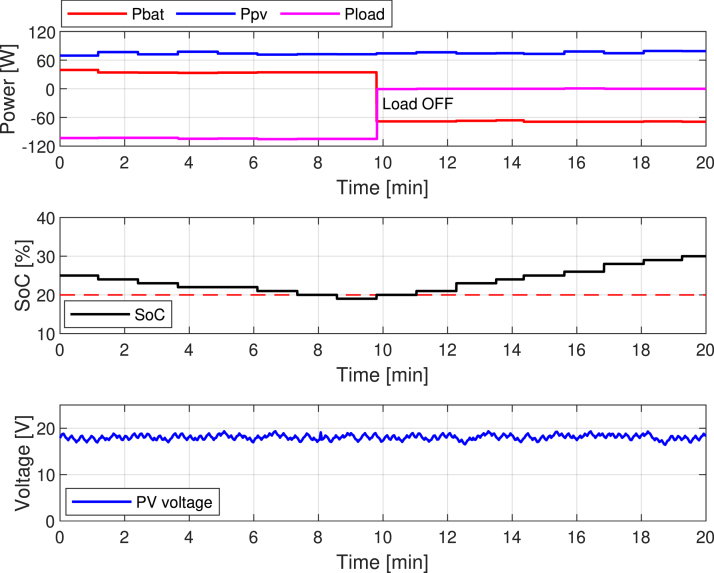
System response under high-load demand.

Fig. 28 shows how the system behaves under a high-load demand scenario. In this case, the PV panel continues delivering its maximum power of approximately 80 W. In comparison, the system establishes a load profile of around 105 W. This imbalance forces the battery to supply the additional 25 W required to meet the total demand.

The top plot of Fig. 28 shows that the $P_{bat}$ begins discharging to compensate for the power deficit. As a result, the middle plot illustrates a continuous decline in the battery’s SOC. The SOC steadily decreases until it reaches the lower allowable limit of 20%, marked by the red reference line. When the system reaches this critical threshold, the PMS activates a protective mechanism to preserve battery health and maintain system stability. Specifically, the PMS deactivates AC and DC loads to reduce total power demand. The top plot marks this event with the annotation ’Load OFF,’ showing a significant drop in $P_{load}$ and a return of $P_{bat}$ to nearly 0 W as the PV panel takes over the reduced load.

After the system disconnects the load, the battery recharges using the PV panel’s surplus energy. The middle plot reflects this recovery phase, showing a gradual increase in SOC after minute 10 as it moves away from the critical 20% threshold. The bottom plot displays the PV panel voltage, which remains relatively stable around 18–20 V throughout the process, ensuring consistent energy production. The MPPT algorithm maintains the PV panel’s optimal operating point, even during dynamic load and battery charging conditions.

## 8 Conclusions

This paper proposes the development of low-power microgrid hardware based on power converters and controllers to carry out the experimental test for the second and third levels of the hierarchical control structure for microgrids. Validations show the system can operate up to 200W of PV panels and loads. Also, the hardware uses low-cost elements and control boards, making the proposed hardware cheaper than commercial hardware with similar specifications.

## CRediT authorship contribution statement

**Cristian Escudero-Quintero:** Writing – original draft, Visualization, Validation, Software. **Juan Pablo Guzman-Rodriguez:** Writing – original draft, Validation, Software, Investigation. **Juan Pablo Villegas-Ceballos:** Writing – review & editing, Validation, Software, Methodology, Investigation, Conceptualization. **Elkin Edilberto Henao-Bravo:** Writing – original draft, Methodology, Conceptualization. **Daniel Gonzalez-Montoya:** Project administration, Methodology, Funding acquisition, Conceptualization.

## Ethics statements

The authors declare that this study did not involve any human subjects or animal experiments; therefore, no ethical approval was required.

## Declaration of competing interest

The authors declare that they have no known competing financial interests or personal relationships that could have appeared to influence the work reported in this paper.
